# Using Recombinant Human Collagen With Basic Fibroblast Growth Factor to Provide a Simulated Extracellular Matrix Microenvironment for the Revascularization and Attachment of Islets to the Transplantation Region

**DOI:** 10.3389/fphar.2019.01536

**Published:** 2020-01-10

**Authors:** Qunyan Zhu, Cuitao Lu, Xuan Jiang, Qing Yao, Xue Jiang, Zhiwei Huang, Yina Jiang, Lei Peng, Hongxing Fu, Yingzheng Zhao

**Affiliations:** ^1^ College of Pharmaceutical Sciences, Wenzhou Medical University, Wenzhou, China; ^2^ Engineering Laboratory of Zhejiang Province for Pharmaceutical Development of Growth Factors, Biomedical Collaborative Innovation Center of Wenzhou, Wenzhou, China; ^3^ Trauma Center, The First Affiliated Hospital of Hainan Medical College, Haikou, China

**Keywords:** diabetes, recombinant human collagen (RHC), basic fibroblast growth factor (bFGF), islet–matrix attachments, islet transplantation

## Abstract

Islet transplantation is considered a potential therapeutic option to reverse diabetes. The pancreatic basement membrane contains a variety of extracellular matrix (ECM) proteins. The abundant ECM is essential for the survival of transplanted islets. However, the ECM proteins necessary for maintaining islet vascularization and innervation are impaired by enzymatic digestion in the isolation process before islet transplantation, leading to destruction of islet microvessels. These are the primary concern and major barrier for long-term islet survival and function. Thus, it is crucial to create an appropriate microenvironment for improving revascularization and islet function to achieve better transplantation outcome. Given the importance of the presence of ECM proteins for islets, we introduce recombinant human collagen (RHC) to construct a simulated ECM microenvironment. To accelerate revascularization and reduce islet injury, we add basic fibroblast growth factor (bFGF) to RHC, a growth factor that has been shown to promote angiogenesis. In order to verify the outcome, islets were treated with RHC combination containing bFGF and then implanted into kidney capsule in type 1 diabetic mouse models. After transplantation, 30-day-long monitoring displayed that 16 mg–60 ng RHC-bFGF group could serve as superior transplantation outcome. It reversed the hyperglycemia condition in host rapidly, and the OGTT (oral glucose tolerance test) showed a similar pattern with the control group. Histological assessment showed that 16 mg–60 ng RHC-bFGF group attenuated apoptosis, promoted cellular proliferation, triggered vascularization, and inhibited inflammation reaction. In summary, this work demonstrates that application of 16 mg–60 ng RHC-bFGF and islets composite enhance the islet survival, function, and long-term transplantation efficiency.

## Introduction

Diabetes is one of the most common serious chronic diseases and is associated with several serious complications, including cardiovascular, kidney, eye, nerve, cerebrovascular, and peripheral vascular diseases (Nathan, 1993; Bader et al., 2016; Wang et al., 2017). Type 1 diabetes (T1D) is an autoimmune-mediated metabolic disease that is characterized by permanent destruction of insulin-producing beta cells, which results in absolute insulin deficiency (Davis et al., 2012). The main therapies for controlling glycemic levels are exogenous insulin administration and oral hypoglycemic agents. Unfortunately, most of the time, these treatments fail to control the blood glucose level, which results in severe hypoglycemia and complications (Bogdani et al., 2014). Islet transplantation offers therapeutic potential for patients with T1D to normalize glucose metabolism and prevent the complications of the disease (Davis et al., 2012). Despite the rapid progress that has been made in islet transplantation after the disclosure of the “Edmonton protocol,” multiple donors are needed to achieve long-term insulin independence, which limits the large-scale application of this protocol. The disruption of islet–matrix attachments or the extracellular matrix (ECM) components between endocrine and exocrine cells during isolation decreases islet function and viability before and after transplantation, which severely hinders the efficiency of transplantation (Barrett-Connor and Orchard, 1985). Furthermore, numerous studies demonstrated that 20~40% of islets undergo apoptosis during the culture. However, pre-conditioning in culture prior to islet transplantation, such as the supplementation of angiogenic agents (Uzunalli et al., 2015; Qiu et al., 2017), antioxidant agents, or oxygen carriers in the culture medium, has enabled islets to obtain longer (Emamaullee et al., 2008; Maillard et al., 2011; Brandhorst et al., 2013; Schaschkow et al., 2015), better graft function and greater efficiency (Kin et al., 2008; Schaschkow et al., 2015).

ECMs, such as collagen, have been developed to address apoptosis during islet culture, to preserve islet morphology, to promote cell survival, and to provide essential physical scaffolding for cellular constituents, which can enhance islet insulin secretion and reinforce islet structure to resist mechanical stress during transplantation (Salvay et al., 2008; Schaschkow et al., 2015). Collagen type III also has been demonstrated to enhance microvascular strength and elasticity, provide adequate nutrients in cells, and directly bind to hemangioblasts, thereby promoting the formation of new blood vessels (Sailakshmi et al., 2011). In this study, recombinant human collagen (RHC), collagen type III, was obtained from a high-density fermented supernatant of GS115/Ppic9KG6 fermentation, which solved the problems of conventional extraction methods, such as poor hydrophilicity and immune rejection. Growth factors, such as basic fibroblast growth factor (bFGF), are natural substances that are capable of stimulating cellular growth, proliferation, healing, and cellular differentiation (Barrientos et al., 2008; Xiang et al., 2011). Our previous study demonstrated that bFGF had potent angiogenetic capabilities in fibroblasts and epithelial cells, which thus promote angiogenesis and are beneficial to islet revascularization (Zhao et al., 2014).

**Figure 1:**
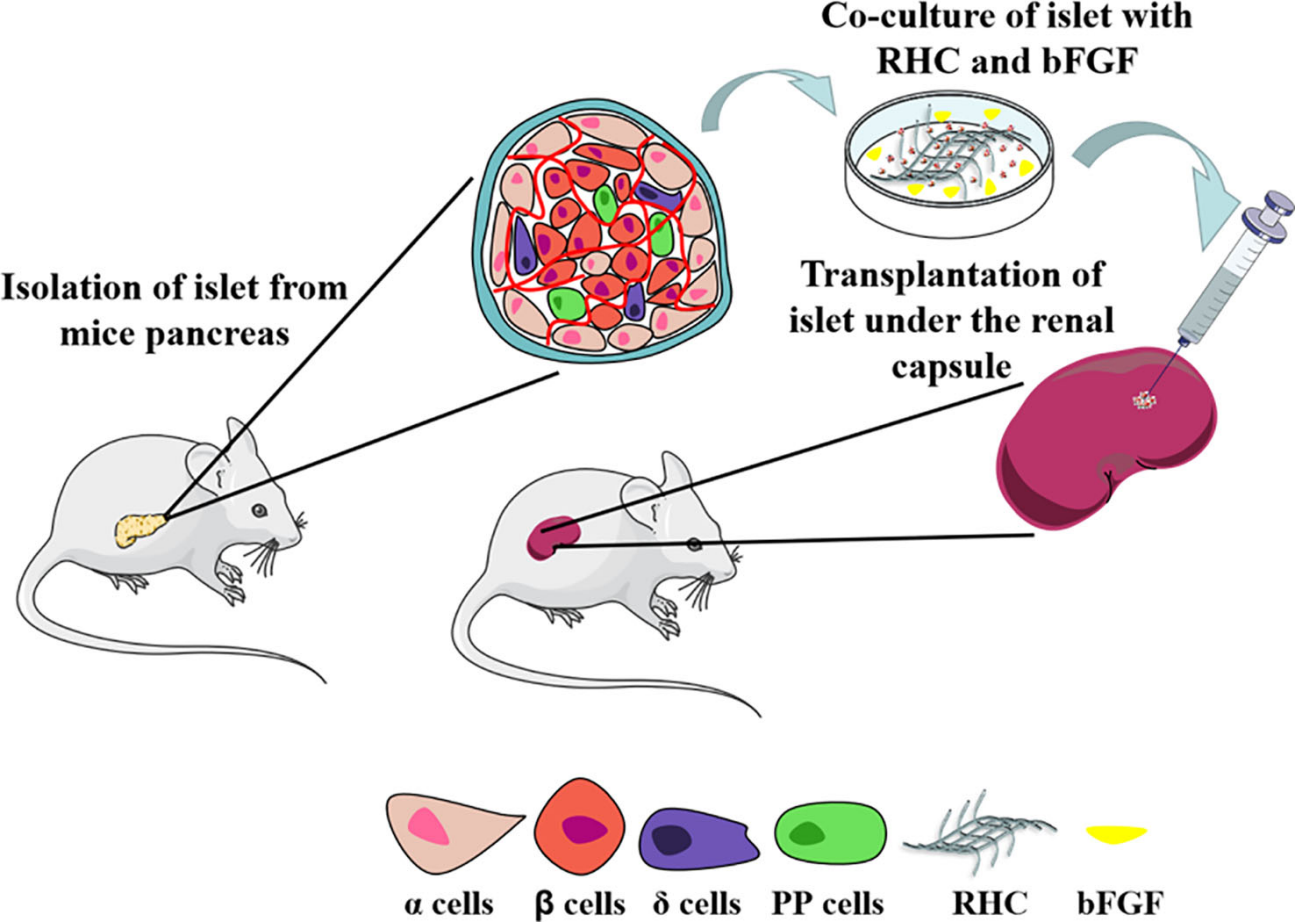
Scheme of RHC-bFGF islet transplantation for reversal type 1 diabetes.

In this study, we investigated a co-culture system of combined bFGF and RHC to provide a simulated ECM microenvironment for islets. We hypothesize that such a microenvironment can rebuild the islet–matrix attachments and, thus, promote the revascularization of islets, which will benefit islet culture both *in vitro* and *in vivo* (as illustrated in **Figure 1**).

## Materials and Methods

### Animals and Reagents

BALB/C male mice that were 8–10 weeks old were obtained from Shanghai, China. All the mice were housed with a 12 h light/dark cycle at 24 ± 1°C and provided with food ad libitum for a week before initiation of the study. All experimental procedures were approved by the Institutional Animal Care and Use Committee of Wenzhou Medical University. Collagenase type V, streptozotocin (STZ), fluorescein diacetate (FDA), and propidium iodide (PI) were purchased from Sigma Inc., St. Louis, MO, USA. bFGF was ordered from Gelusite Biology Technology Company, Zhejiang, China. RHC was kindly provided by Nanjing University of Science and Technology, Jiangsu JL, and Biotech Co., Ltd. Ficoll-1077, Ficoll-1119, Hanks' solution, and CMRL-1066 were purchased from Wenzhou Icelltrans Technologies Co., Ltd., Wenzhou, China.

### Islet Isolation

Islets were isolated from the pancreas of mice according to methods that were modified from other studies (Zmuda et al., 2011). Briefly, the mice were anesthetized by inhaling isoflurane and were placed in the supine position on the dissecting table. The abdomen was sprayed with 75% ethanol, followed by opening it as a U-incision; the entrance of the common bile duct into the duodenum was located and ligated; and 2.5 ml collagenase type V (1 mg/ml, dissolved in cold Hank's solution) was injected into the common bile duct by retrograde intubation. Once the pancreas was perfused, it was removed from the mouse by pulling it free from the points of contact with the intestines, stomach, and spleen. The pancreas was transferred in a 50 ml centrifuge tube. Then, the appropriate amount of collagenase type V was added and subjected to digestion in a 37 ± 0.5°C water bath for 3–5 min with gentle shaking. The digested tissue was washed three times with cold Hank's solution. Subsequently, the islets were purified with a discontinuous density gradient (Ficoll-1119, Ficoll-1077, and Hanks' solution) by centrifuging at 2,000 rpm for 5 min with the brake off. The supernatant containing purified islets was washed three times with cold Hank's solution. If necessary, the islets were further purified by handpicking.

### Islet Counting and Equivalent Calculation

The cultured islets were transferred into a culture dish with 0.5 mm lattices for their quantification under the microscope. The number of islets were counted and calculated to the islet equivalents (IEQs; diameter standardizing to 150 mm) (Lembert et al., 2003).

### Islet Culture

The purified islets were divided into six groups according to different culture conditions: 1) FREE; 2) 60 ng bFGF; 3) 1 mg RHC; 4) 16 mg RHC; 5) 1 mg–60 ng RHC-bFGF; and 6) 16 mg–60 ng RHC-bFGF. Each group contained approximately 200–300 purified islets. In the “FREE” group, the islets were cultured in CMRL-1066 medium supplemented with 10% fetal bovine serum (FBS; Gibco, Invitrogen, Inc., USA) and 1% antibiotics (100 U/ml penicillin, 100 ng/ml streptomycin). In the “60 ng bFGF” group, the islets were cultured in CMRL-1066 medium containing bFGF (60 ng/ml). In the “1 mg RHC” group, the islets were cultured in CMRL-1066 medium containing RHC (1 mg/ml). In the “16 mg RHC” group, the islets were cultured in CMRL-1066 medium containing RHC (16 mg/ml). In the “1 mg–60 ng RHC-bFGF” group, the islets were cultured in CMRL-1066 medium containing RHC (1 mg/ml) and bFGF (60 ng/ml). In the “16 mg–60 ng RHC-bFGF” group, the islets were cultured in CMRL-1066 medium containing RHC (16 mg/ml) and bFGF (60 ng/ml). The concentration of bFGF 60 ng/ml was chosen according to our previous study (Qiu et al., 2017).

### Islet Viability and Reactive Oxygen Species *In Vitro*

After being cultured with the different concentration of RHC (0, 1, 16, and 25 mg/ml) and bFGF (60 ng/ml) in six groups for 24 h at 37°C in a humidified 5% CO_2_, the viability of the islets was analyzed by FDA/PI staining. The ratio of green to green plus red cells provided the percentage of viability. Images were examined and recorded with an inverted microscope (Nikon ECLIPSE Ti-S; Ruikezhongyi, Beijing, China).

To visualize the reactive oxygen species, cultured islets were harvested and washed, followed by incubating with dihydroethidium (DHE, Sigma) for 30 min at 37°C, washing with phosphate-buffered saline (PBS, Gibco), and monitoring by an inverted fluorescence microscope.

### Transmission Electron Microscopy Examination

To determine whether the endocrine granules in the beta cells were preserved or released, the ultrastructure of islets was observed by transmission electron microscopy (TEM) (Daoud et al., 2010). The islets in different groups were collected after culturing for 24 h and were fixed immediately in 2.5% (w/v) glutaraldehyde at 4°C overnight. After dehydration in a series of acetone–water solutions, the islets were embedded in Epon. Semi-thin section and toluidine blue staining were performed to observe the islets locations. Finally, ultra-thin sections of at least six blocks per sample were cut and examined with the electron microscope (H-7500, Hitachi, Ibaraki, Japan).

### The Stability of bFGF in the Culture System

To investigate the stability of bFGF in RHC, the degradation of bFGF *in vitro* was conducted. Three bFGF formulations (60 ng/ml bFGF alone, 60 ng/ml bFGF with 1 mg/ml RHC, and 60 ng/ml bFGF with 16 mg/ml RHC) were prepared and incubated at 37°C. At 0, 12, 24, 48, 72, 120, and 168 h, 100 μl of aliquot was extracted and stored at −80°C before detection. The concentration of bFGF was detected by an ELISA kit.

### Induction of Diabetic Mellitus

Irreversible diabetes was chemically induced in mice by single intraperitoneal injections (IP) of 150 mg/kg STZ. Non-fasting blood glucose levels were measured by using a glucometer (Pro doctor^®^, Tai Doc Technology Corp, Beijing, China) from whole blood samples that were obtained by tail snipping. Mice with two consecutive non-fasting blood glucose levels higher than 22.2 mmol/L were used for transplantation. The diabetic mice were randomly divided into six groups of six. The blood glucose levels of donor mice were also confirmed before islet isolation to verify that they were metabolically normal.

### Islet Transplantation

After 6 h culture, the islets in the six groups were collected and prepared for transplantation. The number of transplanted islets was 200 IEQ for each group. Transplantation was performed as reported (Zmuda et al., 2011; Fu et al., 2016). Mice were anesthetized with isoflurane, and the right back was shaved and sterilized with 75% ethyl alcohol. A small incision was made, and the kidney was exposed. The islets in the syringe were injected slowly and scattered under the kidney capsule. Then, the skin was sutured carefully. It is good to ensure that the kidney is kept moist with saline during the entire process. After transplantation, antibiotics were administered every day for a week to reduce the inflammatory response.

### Function Assessment *In Vivo*

After transplantation, non-fasting blood glucose levels and the weights of animals were measured regularly until the end of the study. The graft was considered to be functional only if the non-fasting blood glucose levels were stably maintained at less than 11.1 mmol/L, with no reoccurrence of hyperglycinemia. An oral glucose tolerance test (OGTT) was conducted on day 30 post–islet transplantation to assess metabolic capacity. Mice were placed in fresh cages with access to water but no food for 12 h. Then, 2 mg/ml glucose in 200 mg/ml solution was administered to the mice orally. Blood glucose was evaluated at time 0 (prior to glucose administration) and then at 20, 40, 60, 90, and 120 min after glucose administration. The area under the curve (AUC) of blood glucose was also calculated.

### Histologic and Immunohistochemical Analyses

At 30 days after transplantation, mice were anesthetized and were transcranially perfused with saline. Kidney with islets were resected and fixed in 4% paraformaldehyde overnight, followed by embedding in paraffin and sectioning at 5 μm thickness. The tissue was dewaxed and rehydrated in xylene and gradient alcohol for hematoxylin and eosin (H&E) staining for the evaluation of general morphology and Masson's trichrome staining for the evaluation of fibrosis. To identify proteins in grafts, immunohistochemistry staining was performed, primary anti-insulin (15848-1-AP, 1:400, Proteintech), anti-HIF-1α (ab1, 1:200, Abcam), anti-Caspase-3 (ab13847, 1:200, Abcam), anti-Ki67 (ab15580, 1:400, Abcam), anti-CD31 (ab182981, 1:200, Abcam), and anti-IL-6 (DF6087, 1:300, Affinity) staining. Digital images were acquired by using a light microscope or confocal microscope.

### Statistics Analysis

All analyses were performed using Graph Pad Prism 7 software (Graph Pad Software, Inc., La Jolla, CA, USA). All results are expressed as the mean ± SD for each group. The one-way ANOVA followed by Tukey's test was for comparing the multi-group data. P-values less than 0.05 were considered statistically significant.

## Results

### Islet Viability *In Vitro*

**Figure 2:**
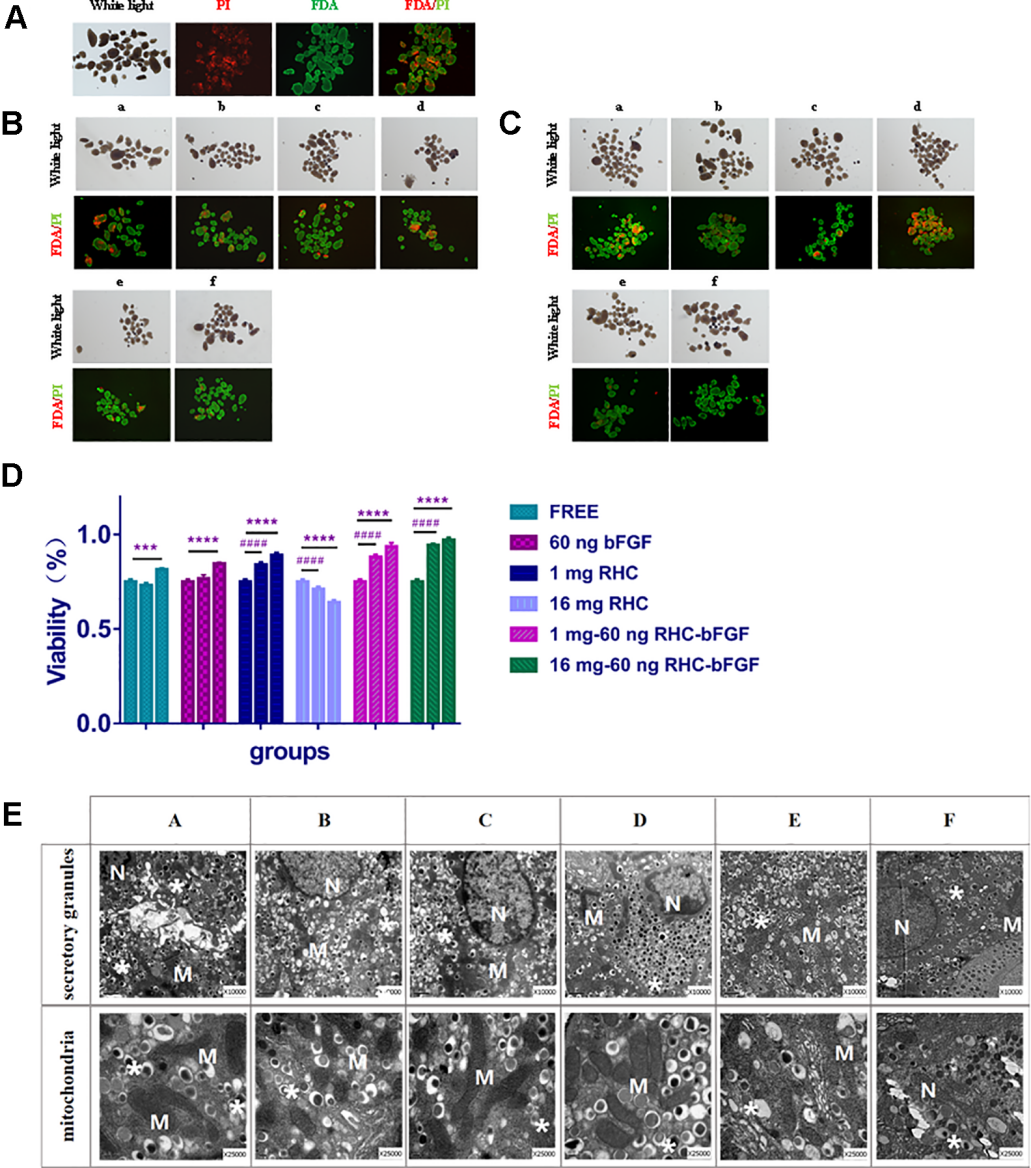
Islet viability and transmission electron microscopy (TEM) results *in vitro*. **(A)** Islet viability at 0 h. **(B)** Islet viability of each group after cultured for 7 h. **(C)** Islet viability of each group after culturing for 24 h. **(D)** Viability statistics after cultivation. The ratio of green to green plus red cells provided the percentage of viability. The three columns of each group represent 0, 7, and 24 h from left to right (****P < 0.0001, ***P < 0.001, 0 vs 24 h; ^####^ *P* < 0.0001, 0 vs 7 h). **(E)** TEM of each group after 24 h culturing.a and A: FREE group; b and B: 60 ng bFGF group; c and C: 1 mg RHC group; d and D: 16 mg RHC group; e and E: 1 mg–60 ng RHC-bFGF group; f and F: 16 mg–60 ng RHC-bFGF group; N: nucleus; M: mitochondria; *: secretory granules.

To calculate the viability of islets under the different conditions, FDA/PI staining was performed at 0, 7, and 24 h after culturing. The results are shown in **Figure 2**.

From the results (**Figure 2**), it can be seen that after 7 h co-culture, the viability of the 1 mg RHC group, the 1 mg–60 ng RHC-bFGF group, and the 16 mg–60 ng RHC-bFGF group increased, while the 16 mg RHC group had the opposite tendency. After culturing for 24 h, the viability of both the 1 mg–60 ng RHC-bFGF group and the 16 mg–60 ng RHC-bFGF group were over 90%, which was better than the viability of the bFGF-alone or RHC-alone groups. We suspected that simultaneous application bFGF and RHC can improve the viability of islets. However, we also found that the viability of the “25 mg RHC” and “25 mg to 60 ng RHC-bFGF” groups was inferior, at either 7 h or 24 h (**Figure 1S**).

The ultrastructure of the islets was observed by TEM (**Figure 2E**). It can be seen that the endocrine granules in the beta cells were preserved, which means that the islets did not degranulate in different culture groups. The mitochondria also did not swell, and the cristae were clear. There was no evidence of membrane disruption in the islets after cultivation for 24 h.

### Islet Hypoxia *In Vitro*

**Figure 3:**
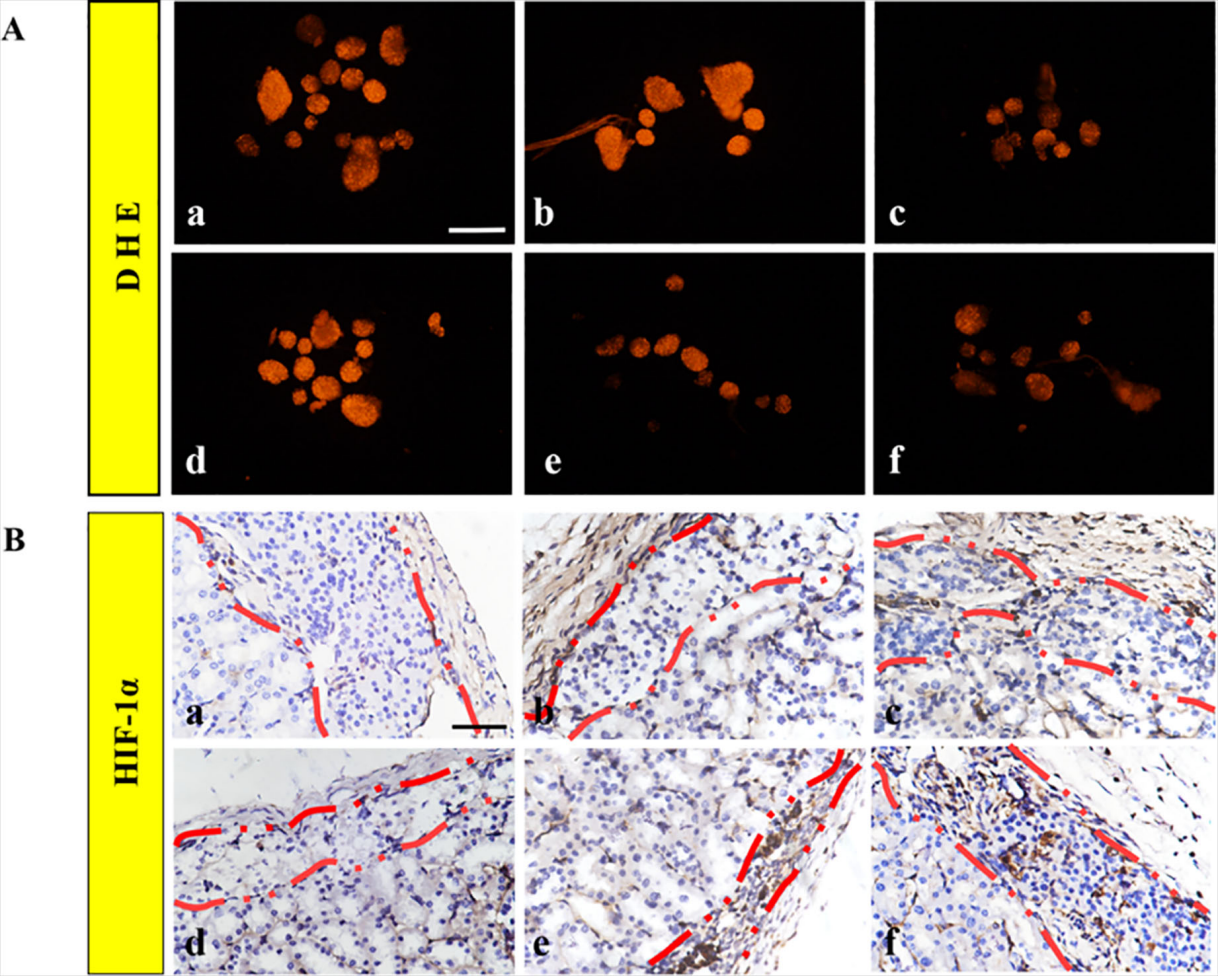
Hypoxia. **(A)** Dihydroethidium (DHE) staining was performed on the islets after 24 h cultivation for all groups. **(B)** Representatives of HIF-1α immunohistochemical staining of the islet graft in each group. a: FREE group; b: 60 ng bFGF group; c: 1 mg RHC group; d: 16 mg RHC group; e: 1 mg–60 ng RHC-bFGF group; f: 16 mg–60 ng RHC-bFGF group.

After co-culturing the islets in each group for 24 h, the fluorescence staining of DHE of the 1 mg RHC group, the 1 mg–60 ng RHC-bFGF group, and the 16 mg–60 ng RHC-bFGF group significantly decreased compared to that of the FREE group, 60 ng group, and 16 mg RHC group (**Figure 3A**), which suggested that the ROS levels significantly decreased when the islets were co-cultured with RHC and/or bFGF, compared with those of the bFGF-alone or FREE islets groups.

HIF-1α was a crucial marker for cellular proliferation in a hypoxic condition (Tsuchiya et al., 2015). After islet transplantation for 30 days, the grafts were removed, and the HIF-1α immunohistochemical staining results are shown in **Figure 3B**. As shown in **Figure 3B**, there was almost no HIF-1α expression in the islets in FREE, and HIF-1α was subtly expressed in the 60 ng bFGF and 16 mg RHC groups. For the 1 mg–60 ng RHC-bFGF group and the 16 mg–60 ng RHC-bFGF group, the expression of HIF-1α had strikingly increased. The previous reports indicated that HIF-1α expression was strongly expressed when the islets were cultured with ECM in a hypoxic condition (Maillard et al., 2011). Based on the results, RHC combined with bFGF would promote cellular proliferation and survival, whereas RHC or bFGF alone would not.

### The Degradation Curve of bFGF

**Figure 4:**
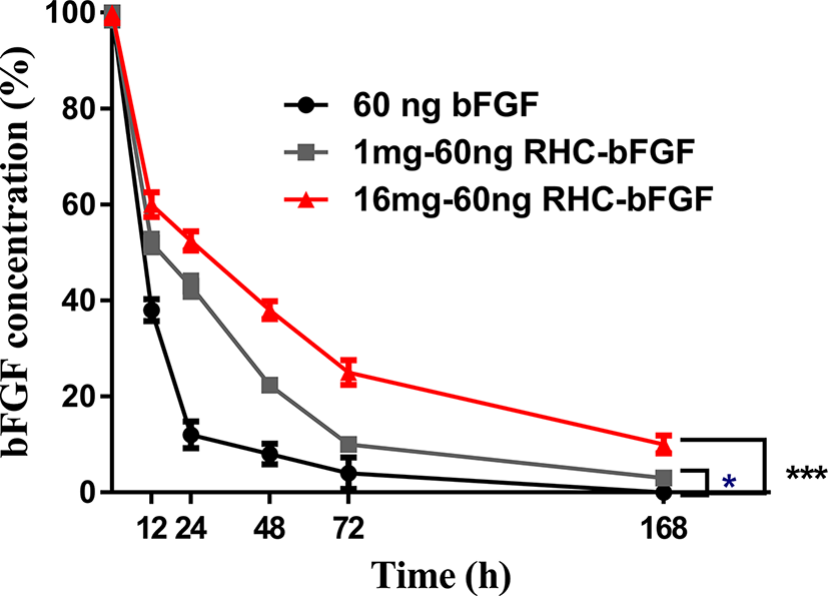
The stability of bFGF with RHC *in vitro* (***P < 0.001, *P < 0.05, compared to 60 ng bFGF).

The impact of RHC on bFGF stability *in vitro* was assessed. The results (**Figure 4**) showed that free bFGF degraded the fastest and decreased to 40% within 12 h. In contrast, the bFGF with the 16 mg RHC group was maintained at approximately 60% bFGF within 12 h. After 72 h, the residual bFGF in the free bFGF group was lower than 5%, while the bFGF concentrations in the 1 mg RHC and 16 mg RHC groups were 10% and 25%, respectively. Even at 168 h, there was still residual bFGF in the 16 mg/ml RHC group. Above all, RHC could increase the stability of bFGF and prolong the action time of bFGF in the islets.

### bFGF With RHC Improved Islet Function *In Vivo*

**Figure 5:**
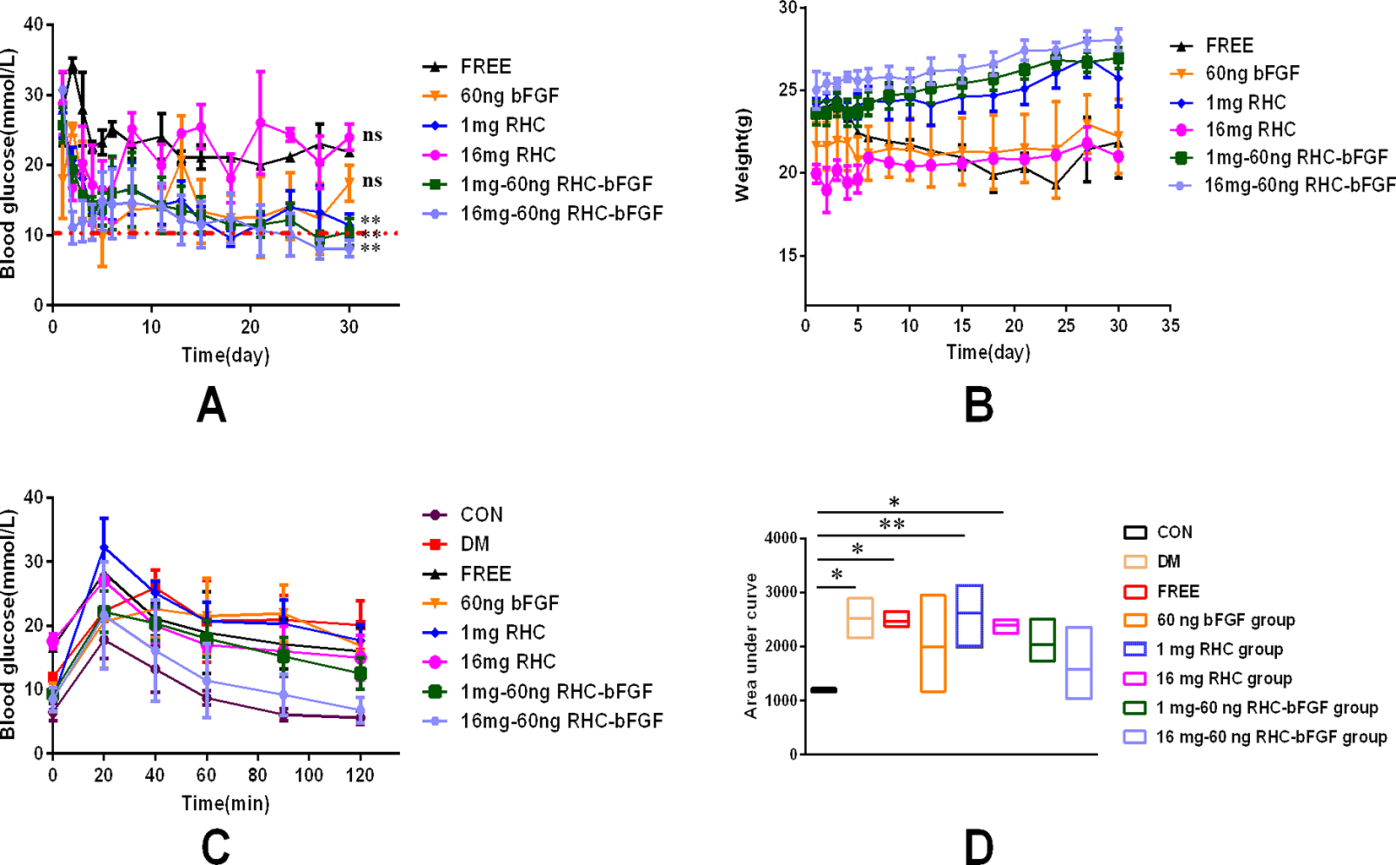
Glucose regulation, body weight, and oral glucose tolerance test (OGTT) after islet transplantation. **(A)** No fasting blood glucose levels from day 0 (day of transplantation) to day 30 post-transplantation. (**P < 0.01, ^ns^P > 0.05, compared to FREE.) **(B)** Change in body weight from day 0 (day of transplantation) to day 30 post-transplantation. **(C)** OGTT of all groups on day 30 post-transplantation. **(D)** Area under the curve (AUC) of corresponding OGTT values for all groups on day 30 post-transplantation. (**P < 0.01, *P < 0.05, compared to CON.)

As shown in **Figure 5A**, the mice transplanted with 200 IEQ 16 mg–60 ng RHC-bFGF islets achieved euglycemia the fastest. Meanwhile, the body weight of the group increased (**Figure 5B**). The body weight of the other two groups (transplanted with 200 IEQ 1 mg RHC islets or 200 IEQ 1 mg–60 ng RHC-bFGF islets) also achieved euglycemia by the end of the experiment (**Figures 5A, B**). However, the other three groups (transplanted with 200 IEQ islets, 200 IEQ 60 ng bFGF islets, or 200 IEQ 16 mg RHC islets) maintained the hyperglycemic condition and exhibited weight loss. Above all, 200 IEQ islets transplanted after culture with 16 mg–60 ng RHC-bFGF achieved the best efficiency, while a previous report demonstrated that 400 IEQ were normally required to achieve the same outcome (Qiu et al., 2017).

To further investigate islet function *in vivo*, an OGTT were performed at 30 days after islet transplantation. As shown in **Figure 5C**, the 16 mg–60 ng RHC-bFGF group shared a similar blood glucose level pattern with that of the normal CON group (**Figure 5C**). Glucose levels in the 1 mg RHC group were significantly higher than those of the 1 mg–60 ng RHC-bFGF group and did not return to the baseline at 120 min. However, the blood glucose levels of the 60 ng bFGF group and the DM group remained at high levels at 120 min after glucose administration. The AUCs of the 1 mg RHC and FREE groups were similar to that of the DM group, while that of the 16 mg–60 ng RHC-bFGF group was similar to that of the CON group (**Figure 5D**). These results suggest that RHC containing bFGF contributed to better islet function.

### bFGF With RHC Maintained Islet Morphology, Enhanced the Reestablishment of the Islet–ECM and Attenuated Apoptosis

**Figure 6:**
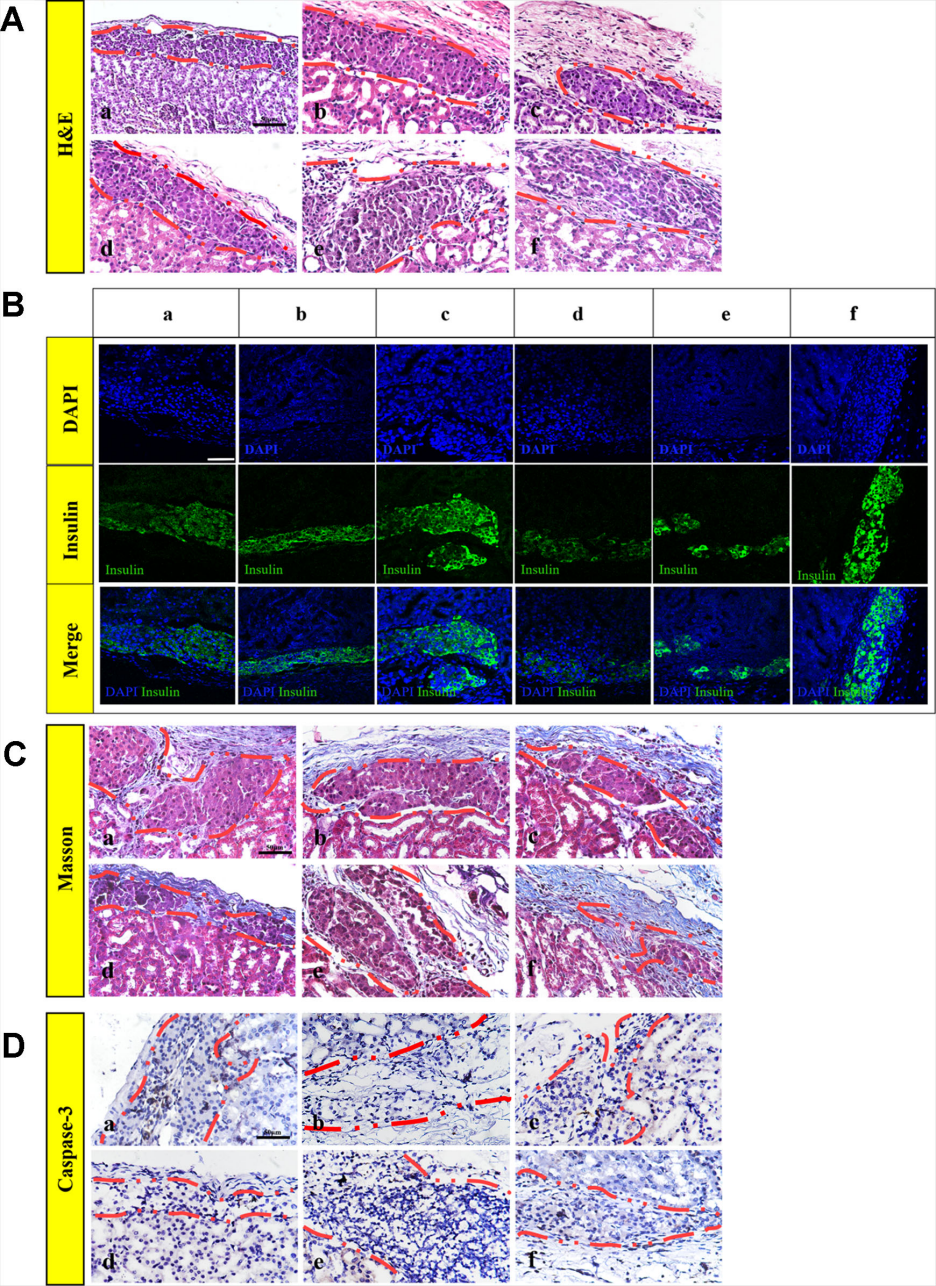
Islet morphology. **(A)** Hematoxylin and eosin (H&E), islets conserved their morphology and integrity on post-transplantation day 30. **(B)** Insulin immunofluorescence, islets secrete insulin after transplantation, particularly 1 mg RHC and 16 mg–60 ng RHC-bFGF groups. **(C)** Masson's trichrome staining of explanted islet grafts on post-transplantation day 30. Masson's trichrome staining showed collagen expression around the transplantation site on day 30 post-transplantation. **(D)** Representative immunohistochemical staining of caspase-3 on post-transplantation day 30. a: FREE group; b: 60 ng bFGF group; c: 1 mg RHC group; d: 16 mg RHC group; e: 1 mg–60 ng RHC-bFGF group; f: 16 mg–60 ng RHC-bFGF group.

The islets had a tendency to form clusters, which can lead to hypoxia and necrosis of the islets and affect the function of the islets (Uzunalli et al., 2015). To monitor the morphology of transplanted islets 30 days after transplantation, (H&E staining (**Figure 6A**), insulin-immunofluorescence staining (**Figure 6B**), and Masson's trichrome staining (**Figure 6C**) were performed on the paraffin section. H&E and positive insulin-immunofluorescence staining showed that the islets were alive, which indicated that the islets remained functional on day 30 post-transplantation. However, as shown in **Figure 6B**, the islets of the FREE and 60 ng bFGF groups gathered and fused into a large cell cluster. There was also a slight aggregation of the islets in group 16 mg RHC, and the islets boundary was blurred. While in the 1 mg RHC, 1 mg–60 ng RHC-bFGF, and 16 mg–60 ng RHC-bFGF groups, the islets had a clear boundary and were single and not clustered, and in the 16 mg–60 ng RHC-bFGF group, the islets were the most intact.

Moreover, collagen is a major component of the ECM, which indicated early attenuation of apoptosis of implanted islets in preliminary studies (Salvay et al., 2008; Davis et al., 2012). Masson's trichrome staining showed collagen existed in the region of transplanted islets of all the groups and collagen was most abundant in the 16 mg–60 ng RHC-bFGF group (**Figure 6C**). Thus, it was speculated that the successful reestablishment of the islet–ECM was crucial for minimizing islet apoptosis and preserving islet survival and function, and in turn, accounted for the better transplantation outcome (Wang and Rosenberg, 1999; Kim et al., 2012; Mao et al., 2017). Hence, it was speculated that bFGF-RHC was crucial in facilitating transplantation efficiency.

In addition, **Figure 6D** shows the caspase-3 immunohistochemistry staining of the graft on day 30 post-transplantation. The FREE group had an obvious positive staining compared with that of the other groups, especially in the central region of the islet clusters, and apoptosis was obvious, which was related to the tendency of the islets to form clusters causing the central region to be susceptible to hypoxia and necrosis (**Figure 6D**). The lack of islet–matrix attachments led to rapid cell death, suggesting that the new contact attenuated apoptosis and the islets mass (Schaschkow et al., 2015). In this work, we utilized RHC and bFGF to achieve this purpose. It was proven that bFGF can promote angiogenesis, which was beneficial to the engraftment of islets (Zhao et al., 2014; Qiu et al., 2017), and ECM was shown to be capable of creating contacts.

### RHC Supplemented With bFGF Facilitated Graft Function by Minimizing the Post-Transplantation Inflammatory Reaction, Enhancing the Vascular Density and Increasing the Proliferation

**Figure 7:**
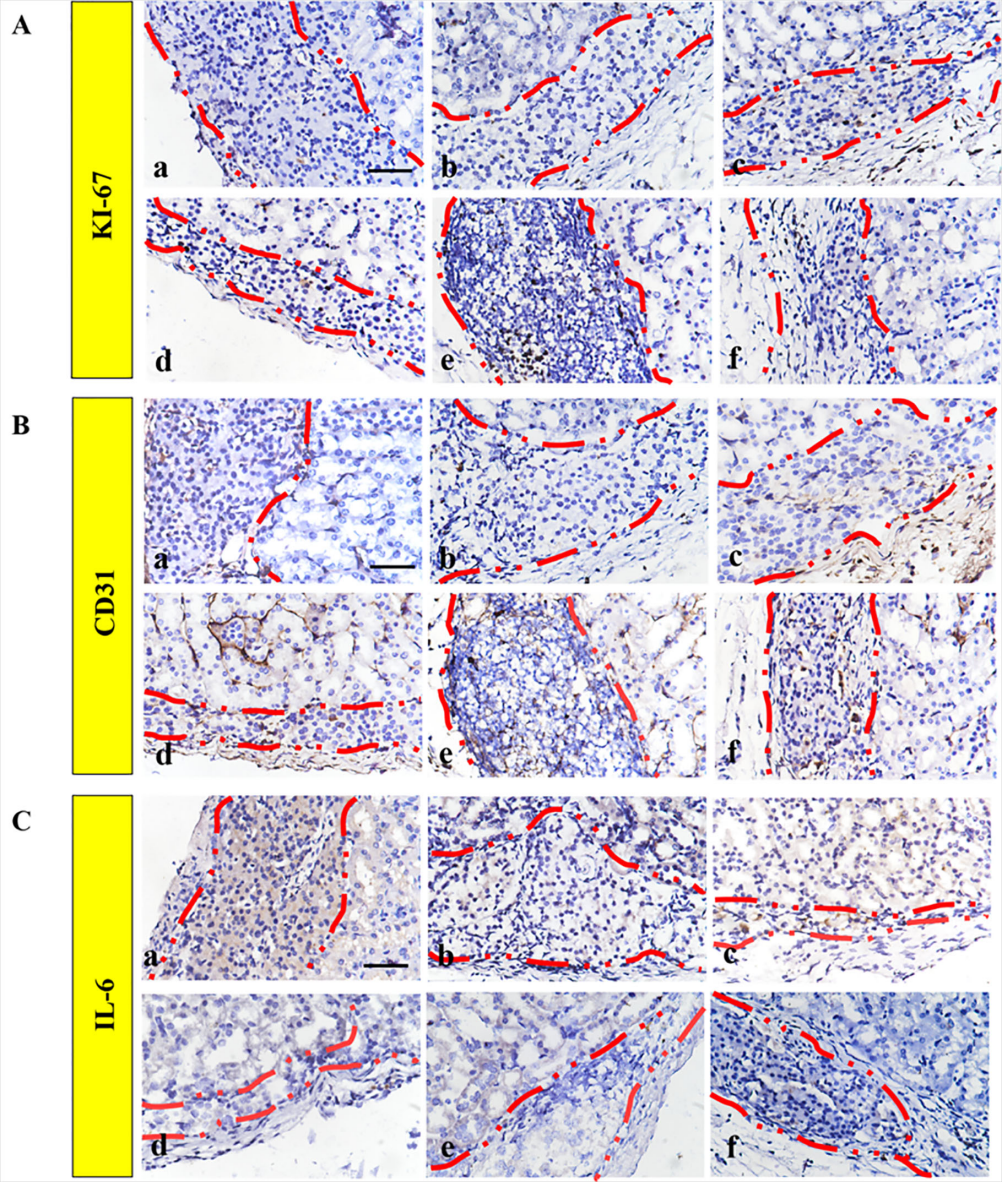
Improved engraftment of transplanted islets by promoting proliferation and angiogenesis. Representative immunohistochemical staining of **(A)** Ki67 and **(B)** CD31 expression. **(C)** IL-6 immunohistochemistry staining was performed to monitor the inflammatory response. a: FREE group; b: 60 ng bFGF group; c: 1 mg RHC group; d: 16 mg RHC group; e: 1 mg–60 ng RHC-bFGF group; f: 16 mg–60 ng RHC-bFGF group.

The ECM proteins could promote cell survival and proliferation, which resulted in the functionality of β-cells (Lai et al., 2005). In addition, Ki67 is a biomarker for evaluating cell proliferation around the graft. **Figure 7A** shows that a low level of Ki67 was found in the FREE group and the 60 ng bFGF group, while significantly higher expression was found in other groups. Moreover, the level of Ki67-positive cells was significantly higher in the 1 mg–60 ng RHC-bFGF group compared with the 1 mg RHC group. These data suggest that RHC alone or RHC supplemented with bFGF facilitated islet proliferation.

The engraftment efficiency was greatly hindered by the destruction of the intra-islet blood vessel network and poor diffusion of nutrients. Therefore, it is critical to address the revascularization issue, which will improve the islet survival and functionality, and ultimately the transplantation outcome (Mao et al., 2017). To investigate the effect of pre-treated islets on islet revascularization, immunohistochemistry staining of CD31 was performed (**Figure 7B**). Based on the results, the density of the CD31 hot point was significantly higher in the high-RHC group than in the low-RHC group in the RHC-alone groups. By adding bFGF, the density of the CD31 hot point of the high concentration group was still higher than that of the low concentration group, and the expression was higher than that of the RHC-alone group, and there was a remarkable difference compared with the levels observed in the bFGF group alone. These data indicate that RHC supplemented with bFGF specifically increased the revascularization after transplantation.

We examined the inflammatory reaction to the implanted islets. IL-6 immunohistochemistry staining revealed that bFGF could inhibit the inflammatory reaction surrounding the implanted islets compared with the staining observed in the FREE group (**Figure 7C**). The RHC-alone group exhibited subtle expression of IL-6. However, IL-6 was almost not observed when RHC was combined with bFGF compared with the expression in the RHC-alone and FREE groups. As previously reported, the 60% of islets that underwent apoptosis during early transplantation was due to the inflammatory reaction (Barshes et al., 2005). In this work, RHC combined with bFGF could minimize the inflammatory reaction to improve islet survival and viability in early transplantation. Eventually, this treatment enhanced the islet transplantation outcome.

## Conclusion

Islet transplantation is the most promising potential therapeutic option for reversing diabetes. However, despite the remarkable progress that has been made to improve the post-transplantation outcome, due to the destruction of islet–matrix attachments and the intra-islet vessel network during the isolation, the reestablishment of islet–matrix attachments and revascularization must be solved to achieve long-term graft function. Herein, we introduce RHC and bFGF into study. RHC is one of the components of the ECM. A previous study showed that the ECM that surrounded the transplanted islet *in vitro* and *in vivo* is a key factor in maintaining vascularization, cell interaction, and the innervations of islets, and further enhances islet survival and function (Salvay et al., 2008; Pepper et al., 2015; Mao et al., 2017). Growth factors are naturally occurring substances that can stimulate cellular growth, proliferation, healing, and cellular differentiation. Our previous study demonstrated that bFGF had potent angiogenetic capabilities in fibroblasts and epithelial cells, thus promoting angiogenesis, which was beneficial in strengthening the graft revascularization (Barrientos et al., 2008; Xiang et al., 2011).

**Figure 8:**
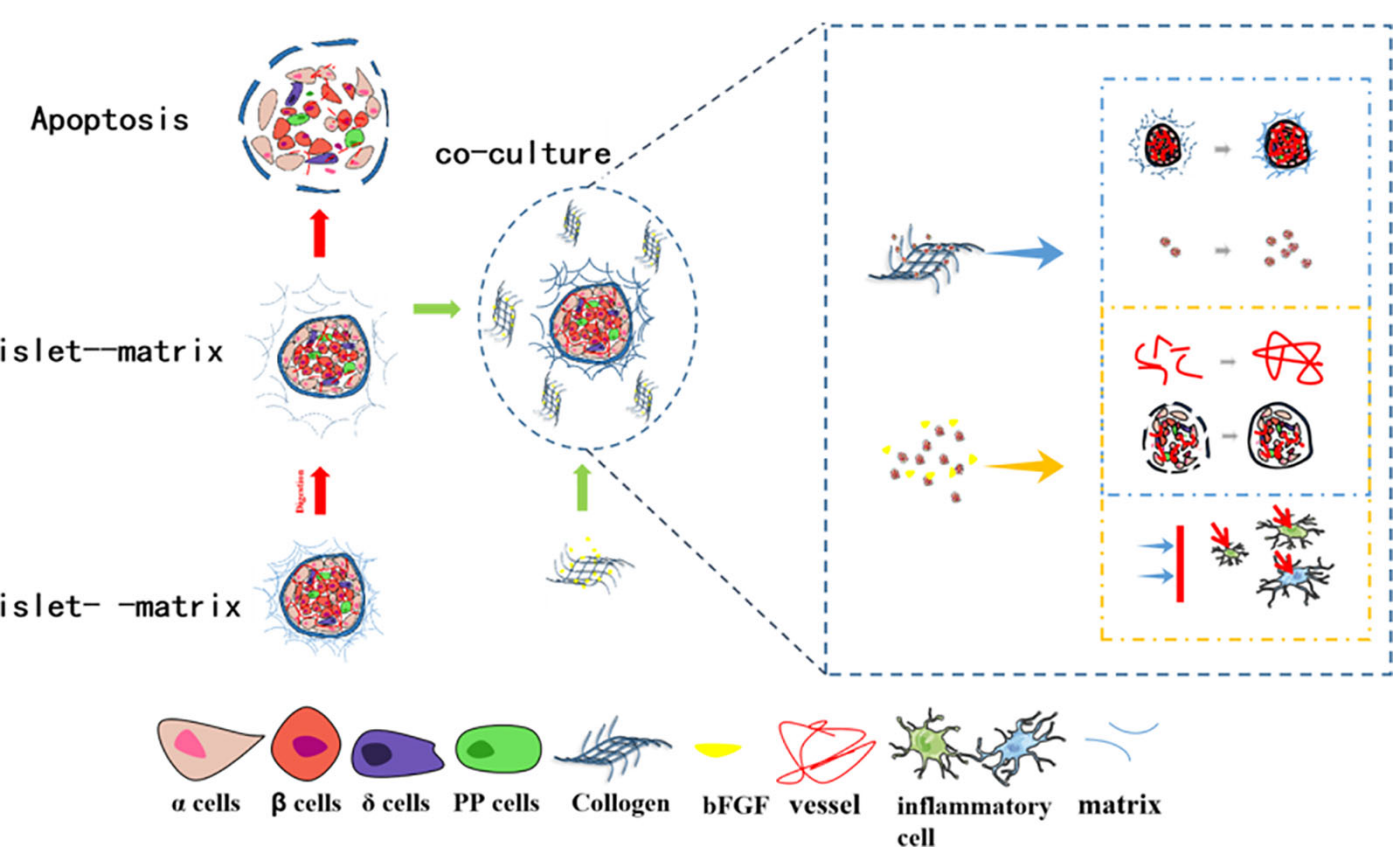
The mechanisms of RHC-bFGF enhanced the post-transplantation outcome to reverse diabetes.

Our study (**Figure 8**) showed that RHC surrounding the islets reconstitutes islet–matrix attachments, promotes islet proliferation, and slows the degradation of bFGF. Meanwhile, bFGF eliminates inflammatory cells around the islet transplanted site. Together, these factors provide a simulated ECM microenvironment for islet survival and act on intra-islet, thereby reconstructing the damaged microvessel. Through a combination of RHC and bFGF, the supply of oxygen and nutrients inside the islets was restored, and apoptosis of the intra-islet was inhibited. The dual effects of RHC and bFGF on the inter-islet and external microenvironment of islets can decrease the quantity of islets needed to reverse diabetes compared with that indicated by the previous study, which, to a certain extent, alleviates the problem of insufficient donors.

Superior transplantation efficiency is evident from the 16 mg–60 ng RHC-bFGF group, which proved to have remarkably better blood glucose responsiveness and blood glucose control. These results suggest that the RHC-bFGF combination enables a simulated ECM microenvironment that facilitates better transplantation efficiency.

## Data Availability Statement

The raw data supporting the conclusions of this article will be made available by the authors, without undue reservation, to any qualified researcher.

## Ethics Statement

The animal study was reviewed and approved by the Institutional Animal Care and Use Committee of Wenzhou Medical University.

## Author Contributions

QZ, CL, YZ, and HF participated in research design. QZ, XuaJ, XueJ, ZH, and YJ were responsible for performing the experiments. QZ, QY, YZ, LP, and HF contributed to the writing and editing of the manuscript. All authors have read and approved this article.

## Funding

This research was supported by the National Natural Science Foundation of China (grant no. 81772316, 81603036, and 81903551), Key Research and Development Program of Zhejiang Province (grant no. 2018C03013), Zhejiang Provincial Natural Science Foundation (grant no. LY19H180001, LQ19H300001, LY17H180008), Zhejiang Provincial Program for the Cultivation of High-Level Innovative Health Talents (Y-ZZ), 151 Talent Project of Zhejiang Province, Zhejiang Provincial Foundation for Health Department (grant no. 2019322086), Wenzhou Municipal Science and Technology Bureau (grant no. Y20190177), and School Talent Start Fund of Wenzhou Medical University (grant no. QTJ15020).

## Conflict of Interest

The authors declare that the research was conducted in the absence of any commercial or financial relationships that could be construed as a potential conflict of interest.

## Supplementary Material

The Supplementary Material for this article can be found online at: https://www.frontiersin.org/articles/10.3389/fphar.2019.01536/full#supplementary-material

Figure 1S Islet viability *in vitro*. Islets were cultured for 7 **(A**, **B)** and 24 h **(C**, **D)**.
