# Supplementary figures and images for: Using Recombinant Human Collagen With Basic Fibroblast Growth Factor to Provide a Simulated Extracellular Matrix Microenvironment for the Revascularization and Attachment of Islets to the Transplantation Region
Source: Front Pharmacol. 2020 Jan 10;10:1536. doi: 10.3389/fphar.2019.01536 (PMC6965329; doi:10.3389/fphar.2019.01536)

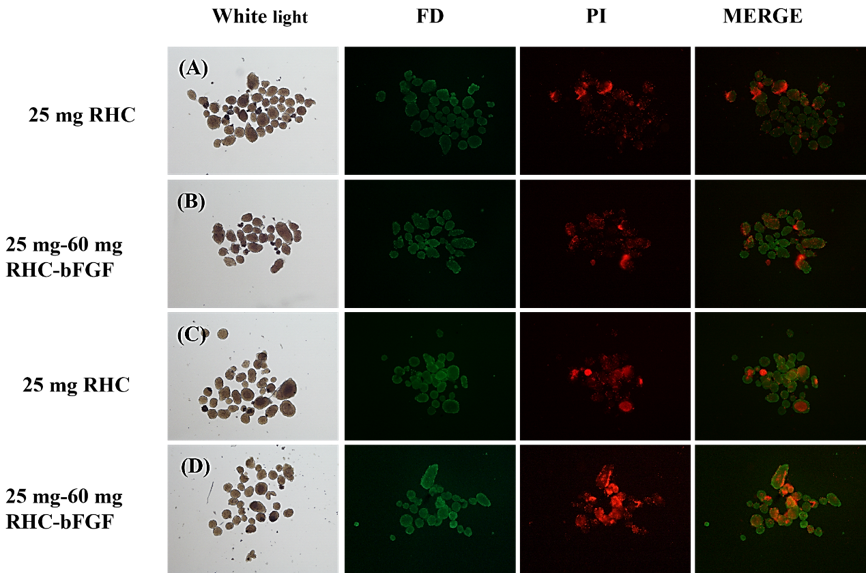

Supplement: Figure 1S — Islet viability in vitro. Islets were cultured for 7 (A, B) and 24 h (C, D). [file Image_1.tif]
